# Therapeutic Botulinum Neurotoxin Ameliorates Motor Deficits and Anxiety, Accompanied by Dopaminergic Neuroprotection and Diminished Microglia Burden in the MPTP-Induced Mouse Model of Parkinson’s Disease

**DOI:** 10.3390/brainsci15090916

**Published:** 2025-08-26

**Authors:** Jerly Helan Mary Joseph, Mercy Priyadharshini Babu Deva Irakkam, Mahesh Kandasamy

**Affiliations:** 1 Laboratory of Stem Cells and Neuroregeneration, Department of Animal Science, School of Life Sciences, Bharathidasan University, Tiruchirappalli 620024, Tamil Nadu, India; josephjerli99@gmail.com (J.H.M.J.); mercyb1999@gmail.com (M.P.B.D.I.); 2 University Grants Commission-Faculty Recharge Programme (UGC-FRP), New Delhi 110002, India

**Keywords:** Parkinson’s disease, Botulinum neurotoxin, MPTP, motor function, anxiety, microglia

## Abstract

**Background**: Parkinson’s disease (PD) is a progressive neurodegenerative disorder characterized by the degeneration of dopaminergic neurons in the substantia nigra (SN), leading to motor impairments and numerous non-motor manifestations, including anxiety. Notably, anxiety has been shown to exacerbate disease progression and hinder treatment outcomes in PD. Botulinum neurotoxin (BoNT), recognized for its ability to block excessive release of acetylcholine (ACh), has been shown to provide clinical effectiveness in managing motor symptoms. BoNT appears to enhance neuroregenerative plasticity and mitigate neuroinflammation through mechanisms speculated to extend beyond its classical mode of action. Nevertheless, reports on its potential anxiolytic and neuroprotective effects in PD remain limited. **Aim**: This study investigated the effect of BoNT on motor and anxiety-like behaviors in a 1-methyl-4-phenyl-1,2,3,6-tetrahydropyridine (MPTP)-induced mouse model of PD. **Methods**: The experimental animals were assessed for behavioral changes using the open field test (OFT), rotarod, pole test, light-dark box test (LDBT), and elevated plus maze (EPM). Immunohistochemistry was employed to enumerate tyrosine hydroxylase (TH)-positive dopaminergic neurons and ionized calcium-binding adapter molecule (Iba)-1 expressing microglia in SN. **Results**: BoNT treatment markedly alleviated motor deficits and anxiety. Quantification of TH- and Iba-1-positive cells revealed that BoNT promotes neuroprotection and minimizes microglial burden in the SN of the PD model. **Conclusions**: The outcome of the study represents the anxiolytic, neuroprotective, and microglial modulatory potentials of BoNT in PD, supporting its therapeutic promise beyond the management of motor symptoms. Given its multifaceted properties, BoNT can be considered a potential therapeutic candidate for PD and other neurological disorders.

## 1. Introduction

Parkinson’s disease (PD) is the second most prevalent neurodegenerative disorder, primarily characterized by the degeneration of dopaminergic neurons in the substantia nigra (SN), a brain region critical for motor functions [1]. According to the global burden of disease (GBD) study, over 11 million individuals were estimated to be affected by PD in 2021 [2]. Considering its sporadic nature and the increase in life expectancy of the global population, approximately 1 in 1,000 individuals aged 60 years and above are affected by PD [2,3]. The prevalence of PD is projected to reach over 25 million cases by 2050 [2]. Compared to women, men appear to be at a higher risk of developing PD, often with an earlier age of onset [4]. Clinically, PD is characterized by both motor and non-motor symptoms. The consequence of dopamine depletion in PD leads to the prominent motor symptoms, such as tremors, rigidity, and bradykinesia, along with a wide range of non-motor symptoms [5,6]. The non-motor symptoms of PD include olfactory dysfunction, cognitive deficit, sleep disorder, personality deformities, autonomic dysfunction, pain, fatigue, depression, and anxiety [7,8]. Among the non-motor manifestations of PD, anxiety is regarded as one of the most debilitating yet frequently underestimated complications, with a substantial impact on disease burden and progression [9,10]. Anxiety-related pathogenic alterations have been recognized as parallel contributors to disease progression in PD, exacerbating clinical symptoms, impairing therapeutic efficacy, increasing mortality risk, and thereby imposing a considerable burden on both the healthcare system and caregivers [11,12,13]. Despite significant progress made in the pharmacological management of motor symptoms, treatment strategies to alleviate anxiety remain largely deprioritized in PD as the underlying pathomechanisms, potential impact, and therapeutic targets remain ambiguous. Given its comorbid nature, multifactorial origins, and limited mechanistic understanding, anxiety in PD has drawn growing research interest. Emerging evidence highlights neuroinflammation as a pivotal pathomechanism, potentially bridging neurodegeneration of dopaminergic neurons in SN and the development of anxiety in PD [14]. In PD, dopaminergic neurons in the SN are vulnerable to proinflammatory cytokines and reactive oxygen species (ROS) released by activated microglia [15]. Eventually, excessive release of proinflammatory molecules from activated microglia has been implicated in the pathogenesis of anxiety across a wide range of neurodegenerative disorders, including PD [16]. Therefore, therapeutic strategies that modulate microglial activation and attenuate neuroinflammation may offer benefits in protecting dopamine neurons from neurodegeneration and mitigating anxiety.

Botulinum neurotoxin (BoNT) produced by *Clostridium botulinum*, a Gram-positive, anaerobic, spore-forming bacterium, has been identified as a potent flaccid paralytic agent that acts by inhibiting the release of acetylcholine (ACh) from presynaptic neurons [17]. Notably, abnormal discharge of ACh and chronic neuroinflammation are observed in aging and various neurological conditions. Due to its neuromodulatory and anti-inflammatory properties, low-dose pharmacological grade BoNT is extensively employed as a therapeutic intervention for neuromuscular and movement disorders, including PD [18]. A substantial scientific body of evidence supports the efficacy of BoNT in alleviating movement deficits in various neurological conditions [18,19]. Notably, BoNT has been demonstrated to be effective in treating tremors, muscle rigidity, involuntary eyelid movements, spasticity, and various forms of dystonia in PD [19,20]. A single dose of BoNT has been shown to produce sustained effects lasting a minimum of 3 to 6 months and in many neurotherapeutic and aesthetic regimens, its benefits can extend up to 1 year or longer [21]. However, in some cases, additional injections are required depending on the required treatment objectives and clinical response of the affected individual. Increasing scientific attention has been directed toward elucidating the signaling mechanisms of BoNT within sensory–motor pathways, particularly in the context of movement disorders such as PD [22,23]. Recent research highlights that beyond its well-known inhibitory effects on cholinergic transmission, BoNT also modulates biochemical pathways associated with fever, pain, inflammation and apoptotic pathways [23,24]. Neuroimmune dysregulation, oxidative stress, and aberrant neuromodulation are increasingly recognized as central mechanisms driving anxiety in aging, which in turn exacerbates the progression and symptomatology of neurodegenerative diseases such as PD [25,26,27]. Furthermore, ample experimental evidence indicated that BoNT exerts neuroprotective effects by enhancing the activity of antioxidant enzymes in both aging and disease models [28]. Despite reports of BoNT-mediated effects on motor symptoms, its anxiolytic properties and protective effects on dopaminergic neurons in PD remain largely unexplored. As PD has been primarily linked to dopamine deficiency resulting in motor impairments and anxiety, the therapeutic potential of ACh-modulating drugs in PD has been largely disregarded. In the physiological state, dopamine exerts an inhibitory effect on the cholinergic system. Thus, dopamine deficiency resulting from neurodegeneration in PD can lead to increased ACh levels, potentially causing a cholinergic crisis in the brain [8]. The cholinergic imbalance in PD could be hypothesized to induce microglial activation, leading to neuroinflammation and hormonal changes associated with anxiety. Therefore, ACh modulatory agents such as BoNT could be highly beneficial not only for neutralizing neurotransmitter imbalance but also for potentially reducing neuroinflammation and providing neuroprotection, thereby alleviating both motor and anxiety-related symptoms. Moreover, identifying the effect of BoNT on microglial modulation and its related pathogenic changes associated with anxiety would provide novel insights and therapeutic advancements. Therefore, this study was conceived to investigate the effects of BoNT on motor and anxiety-like behaviors and examine its outcome on the number of dopaminergic neurons and microglial cells. Based on the observations reported in this study, it can be postulated that BoNT may mitigate neuroinflammation by modulating microglial activation, thereby suppressing the release of proinflammatory mediators. This effect may underlie the possible neuroprotective measures, ultimately leading to improved motor function and reduced anxiety-related behaviors in the animal model of PD.

## 2. Materials and Methods

### 2.1. Experimental Animals and Drug Administration

Male C57BL/6J mice aged 3 months were obtained from Liveon Biolabs, Bangalore, and maintained for the next 30 days in the central animal facility of the university, under a 12-h light/dark cycle at a room temperature of 22–25 °C, with free access to feed and water. At the age of 4 months, the experimental mice (*n* = 36) were divided into four groups as follows: group-1; control (*n* = 9), group-2; BoNT (*n* = 9), group-3; PD (*n* = 9) and group-4; PD + BoNT (*n* = 9). Mice in groups 3 and 4 were administered an intraperitoneal injection of 1-methyl-4-phenyl-1,2,3,6-tetrahydropyridine (MPTP) 30 milligram/kilogram body weight (mg/kg BW) (Sigma, St. Louis, MO, USA, M0896) dissolved in 200 µL of sterile saline for 5 consecutive days, while mice in groups 1 and 2 were injected with the same volume of saline. Eight days following the first injection of MPTP, groups 2 and 4 were administered a single intramuscular injection of BoNT (Allergan, Dublin, Ireland) at a dose of 1 U/kg BW reconstituted in 250 µL of sterile saline. Similarly, the mice in groups 1 and 3 received the same volume of saline. The doses and duration of MPTP and BoNT injections were adopted from previous reports based on the PD-inducing effects of MPTP and the neuromodulating efficacy of BoNT, respectively [28,29]. After 2 weeks of BoNT injection, all experimental mice were subjected to behavioral tests such as open field test (OFT), rotarod test, pole test, light and dark box test (LDBT), and elevated plus maze (EPM). Firstly, all animals were acclimatized in the behavioral room. The lighting in the behavioral room was adjusted appropriately and a video camera was set up above the center of the behavioral equipment. The SMART 3.0 video tracking system (Panlab, Harvard Apparatus, Barcelona, Spain), connected to a semi-automated computer system, was used for recording and digital tracking of animal behavioral experiments. The identity of the animals and treatment groups was blind-coded. The treatment groups and identity of the animals were masked from the experimenter and animals in all groups were randomly assigned to the behavioral tests. Following treatment, animals were subjected to motor behavioral assessments from days 23 to 31, anxiety-related evaluations from days 32 to 37, and cognitive function tests from days 38 to 50. To note, while outcomes of learning and memory were reported by Joseph and Kandasamy, the present study extended and focused on motor functions and anxiety-related behaviors [30].

**Figure 1:**
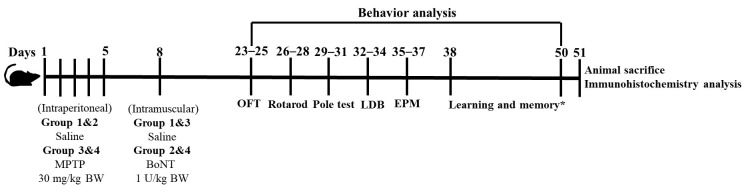
The figure represents the experimental timeline of four groups of mice. The study begins with a series of injections: intraperitoneal injections of either saline or MPTP (30 mg/kg BW) groups from day 1 to 5, followed by intramuscular injections of saline or BoNT (1 U/kg BW) on day 8. From day 23 to 50 a comprehensive behavior analysis is conducted, including the open field test (OFT), rotarod test, pole test, light-dark box test (LDBT), and elevated plus maze (EPM). Upon completion of behavioral experiments, animals were sacrificed for subsequent immunohistochemistry and biochemical analysis. * The outcome of BoNT on learning and memory has been reported by Joseph and Kandasamy [30].

On completion of the behavioral experiments, the mice *n* = 6 per group were anesthetized and perfused as previously described [30]. The brains were collected and subjected to cryosections followed by immunohistochemical assessments. The remaining mice (*n* = 3) from each group were sacrificed and the brains were collected and preserved for biochemical analyses and future studies (Figure 1). All experimental procedures were carried out in accordance with the approval of the Institutional Animal Ethical Committee (IAEC), Bharathidasan University, under the regulations of the Committee for the Purpose of Control and Supervision of Experiments on Animals (CPCSEA), India (Ref. No: BDU/IAEC/P26/2021).

### 2.2. Open Field Test

OFT was used to evaluate exploration-based anxiety-like behaviors in experimental mice. The apparatus consists of a standard wooden arena (120 cm × 120 cm) with 16 grids, each 30 cm in size. The arena was digitally subdivided into an outer zone and an inner zone using SMART 3.0. The outer zone was marked by a red layout and the inner zone was marked by a blue quadrangle. Each mouse was released in the middle of the OFT arena and allowed to move without any interruption. Three trials were conducted for 3 consecutive days and each lasting 5 min. During the trial, parameters like time spent in the outer zone and inner zone by the experimental mouse were recorded using SMART 3.0. At the end of each trial, the mouse was released back to its home cage and the entire arena was sterilized with 70% ethanol.

### 2.3. Rotarod Test

Motor coordination and balance were assessed using a semi-automated rotarod apparatus (Inco, Ambala, India). Mice were pre-trained on the rotating rod at 3 revolutions per minute (RPM) for 3–5 min 1 day prior to testing to acclimatize to the apparatus. On the test day, each mouse was placed individually on the rotating rod, which gradually accelerated from 3 to 25 RPM within 5 min. The latency and the RPM at which animals fall were recorded for each trial. Each animal underwent three trials, with at least 30 min of rest between trials to prevent exhaustion. The average latency to fall and the speed at which the mouse fell were calculated as measures of motor performance. While the reduction in latency indicates impaired motor coordination and balance, increased endurance on the rotarod is considered an improvement in motor function.

### 2.4. Pole Test

The pole test was used to evaluate the motor coordination and balance of each mouse in the experimental groups. A vertical pole, 60 cm in height and 1.9 cm in diameter, equipped with a solid platform, was utilized in this study. The base of the pole was positioned inside a clean cage filled with fresh husk bedding. Each mouse was gently positioned at the top of the pole in a head-upward orientation. The time taken to turn downward (T-turn) and the total time to descend to the base (T-total) were recorded. Each animal underwent three trials with a minimum interval of 30 min between each trial.

### 2.5. Light and Dark Box

LDBT was used to assess preference-based anxiety-like behavior in mice, considering their natural aversion to illuminated areas. The LDB apparatus has two compartments, a closed dark and an open light compartment. While the dark compartment was digitally marked with a red outline, the light compartment was marked with a blue outline using SMART 3.0. Each mouse was released in the middle of the light compartment and allowed to freely access the entire apparatus. Three trials were conducted for 3 consecutive days and each trial was conducted for 5 min. The time spent by each mouse in light and dark compartments was tracked using SMART 3.0. The whole LDB apparatus was wiped with 70% ethanol and dried.

### 2.6. Elevated Plus Maze

EPM was used to analyze acrophobia-related anxiety in experimental mice. The EPM consists of 4 arms with 2 unprotected open arms of size 50 cm × 10 cm and 2 closed arms of size 50 cm × 10 cm protected by 30 cm high sidewalls. The four arms were digitally designated with different colors using SMART 3.0. The open arms (OA) were marked with green and yellow and the closed arms (CA) were marked with blue and violet colors. Each mouse was released at the center and allowed to move freely. Three trials were conducted over three consecutive days, with each trial lasting 5 min. The number of entries and time spent in the OA and CA by each mouse were estimated using SMART 3.0. At the end of the trial, the mouse was released back to its cage and the apparatus was cleaned with 70% ethanol and allowed to dry.

### 2.7. Perfusion of Experimental Mice

On completion of the behavioral experiments, mice in all groups were anesthetized by intraperitoneal injection of ketamine (85 mg/kg BW)/xylazine (10 mg/kg BW) and transcardially perfused with 0.9% sodium chloride (NaCl) (Sisco Research Laboratories (SRL), Mumbai, India), followed by 4% paraformaldehyde (PFA) (Himedia, Mumbai, India). The brains were dissected and stored in 4% PFA at 4 °C for 24 h. Later, the brains were preserved in 30% sucrose (SRL, Mumbai, India) at 4 °C. The brains were cryosectioned using a sliding microtome (Weswox, Haryana, India). The two hemispheres of the brain were separated and placed on the block holder of the sliding microtome. The brain was embedded with PolyFreeze tissue freezing medium (Sigma-Aldrich, St. Louis, MO, USA) and allowed to freeze using dry ice. Once the brains were completely frozen, sagittal sections of 30 μm thickness were carried out and serially collected in 12 tubes containing cryoprotectant solution and stored at −20 °C for further analyses.

### 2.8. Immunohistochemistry

One out of every twelve brain sections (30 µm thickness, 360 µm apart) was processed for TH and Iba-1 staining, followed by microscopic examination. The two sets of free-floating brain sections were taken in 12-well plates (Tarson, Kolkata, India), and washed thrice in 1x Tris-buffered saline (TBS) for 10 min each on an arbitrary shaker (Tarson, Kolkata, India). Then, the sections were incubated with 10 mM sodium citrate buffer for antigen retrieval. After an additional three washes in 1x TBS for 10 min each, the brains were blocked for 1 h in 3% bovine serum albumin (BSA) (HiMedia, Mumbai, India). One set of brain sections was incubated with rabbit anti-tyrosine hydroxylase antibody (Cell Signaling Technology, Danvers, MA, USA; dilution 1:250), while another set was treated with rabbit anti-ionized calcium-binding adapter molecule 1 (Iba-1) antibody (Cell Signaling Technology, Danvers, MA, USA; dilution 1:250) for 48 h at 4 °C. Then brains were washed thrice with 1x TBS for 10 min and then incubated for 24 h at 4 °C with goat anti-rabbit Dylight Red 594 (Novus Biologicals, Littleton, CO, USA; dilution 1:500). Later, the sections were washed thrice with 1x TBS for 10 min. After 5 min of incubation with 0.1 mg/mL of 4′,6-diamidino-2-phenylindole (DAPI) (Himedia, Mumbai, India), the sections were again rinsed with 1x TBS for 10 min. The brain sections were placed onto the microscopic glass slides (Borosil, Mumbai, India) and dried overnight in the dark. The sections were then mounted with ProLong^TM^ Glass antifade mountant (Thermo Fisher Scientific, Waltham, MA, USA). The counting procedures were performed blinded on coded slides and examined using a fluorescence microscope (DM750, Leica Microsystems, Wetzlar, Germany) connected to a computer installed with ImageJ plugin with a cell counter (version number: 1.53k). The area of the pars compacta of SN was marked in each brain section using ImageJ software. Immunopositive cells were quantified on five non-overlapping brain sections per animal using the ImageJ cell counter plugin. The average cell count was then calculated for each mouse, as previously described [31]. The microscopic images were captured at 400× magnification and representative images are presented in the respective figures of the results section.

### 2.9. Statistical Analysis

Data were represented as mean ± standard error mean (SEM). Statistical significance was determined with a one-way analysis of variance (ANOVA) and further analyzed by Tukey’s post hoc test for multiple comparisons using GraphPad Prism 5 software. *p* < 0.05 was considered to be statistically significant.

## 3. Results

### 3.1. BoNT Improved Locomotion and Reduced Anxiety-Related Behavior in MPTP-Induced Experimental Mice in the Open Field Test

**Figure 2:**
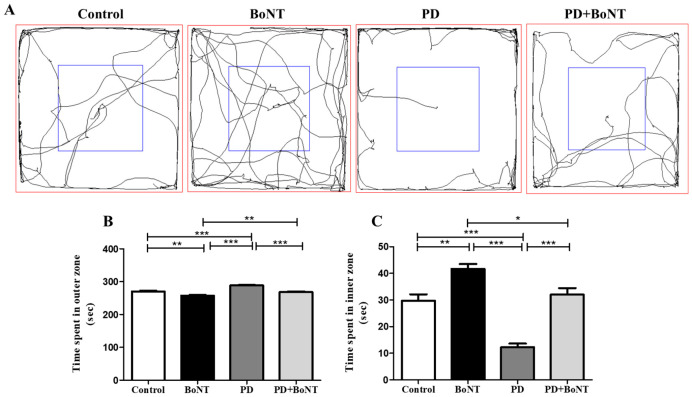
BoNT reduces explorative-based anxiety-like behavior in OFT in MPTP-induced experimental mice. (**A**) Representative tracking image of a mouse during OFT from Control (*n* = 9), BoNT (*n* = 9), PD (*n* = 9), and PD + BoNT (*n* = 9). The outer zone is marked by a red square and the inner zone is marked by a blue square. The bar graph represents (**B**) time spent in the outer zone, and (**C**) time spent in the inner zone by the experimental mice. Statistical significance was determined with one-way ANOVA and further analyzed by Tukey’s post hoc test for multiple comparisons (*, **, *** indicates *p* value ≤ 0.05, ≤0.01, and ≤0.001, respectively).

In OFT, the difference in the time spent by mice among experimental groups in the outer and inner zones was evaluated. Mice in the PD group spent more time in the outer zone when compared to mice in control, BoNT, and PD + BoNT groups (outer zone; Control = 270 ± 2; BoNT = 258 ± 2; PD = 288 ± 2; PD + BoNT = 268 ± 2, *** *p* < 0.0001). The experimental mice treated with BoNT spent more time in the inner zone compared to mice in control, PD, and PD + BoNT mice (inner zone; Control = 30 ± 2; BoNT = 42 ± 2; PD = 12 ± 2; PD + BoNT = 32 ± 2, *** *p* < 0.0001). Considering that less anxious animals tend to explore the central areas of the test arena, the OFT results suggest that BoNT treatment reduces anxiety in MPTP-induced experimental mice (Figure 2). With reference to motor function, our recent report demonstrated that the number of grids crossed and the distance traveled were reduced in the PD group compared to the other groups, whereas BoNT-treated animals exhibited an increased number of grid crossings and more distance traveled. Moreover, the grid crossings and distance traveled by the PD + BoNT group were markedly improved compared to the PD group, in association with increased neurogenesis in the hippocampus [30].

### 3.2. BoNT Improves Motor Coordination and Balance in MPTP-Induced Experimental Mice in the Rotarod Test

**Figure 3:**
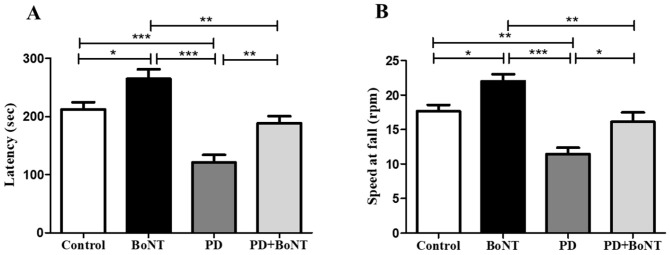
BoNT improves motor coordination performance in MPTP-induced PD mice in the rotarod test. The bar graph represents (**A**) the latency to fall in Control (*n* = 9), BoNT (*n* = 9), PD (*n* = 9), and PD + BoNT (*n* = 9) and (**B**) the speed at fall in experimental mice. Statistical significance was determined with one-way ANOVA and further analyzed by Tukey’s post hoc test for multiple comparisons (*, **, *** indicates *p* value ≤ 0.05, ≤0.01, and ≤0.001, respectively).

In the rotarod-based evaluation of motor coordination and balance, mice in the PD group exhibited a significant reduction in latency to fall, indicating that they tended to fall more easily compared to the control, BoNT, and PD + BoNT groups (latency: Control = 212 ± 12; BoNT = 265 ± 16; PD = 121 ± 13; PD + BoNT = 188 ± 12; *** *p* < 0.0001). Regarding the RPM at the time of fall, mice in the PD group fell at significantly lower speeds, reflecting impaired balance and reduced coordination compared to the other groups (speed at fall: Control = 18 ± 1; BoNT = 22 ± 1; PD = 11 ± 1; PD + BoNT = 16 ± 1; *** *p* < 0.0001). Mice administered BoNT alone displayed a marked increase in latency and remained longer on the rotating rod, even at higher RPMs, compared to the control, PD, and PD + BoNT groups. Furthermore, mice in the PD + BoNT group showed notable improvements in latency and performed better at higher speeds than those in the PD group (Figure 3).

### 3.3. BoNT Restores Motor Functions in MPTP-Induced Experimental Mice in the Pole Test

**Figure 4:**
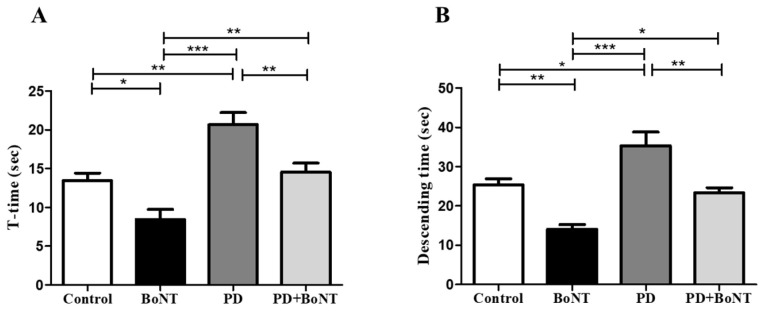
BoNT improves motor performance in MPTP-induced experimental mice in the pole test. The bar graph represents (**A**) time taken to turn (T-time) in Control (*n* = 9), BoNT (*n* = 9), PD (*n* = 9), and PD + BoNT (*n* = 9) and (**B**) time taken to descend downwards in experimental mice. Statistical significance was determined with one-way ANOVA and further analyzed by Tukey’s post hoc test for multiple comparisons (*, **, *** indicates *p* value ≤ 0.05, ≤0.01, and ≤0.001, respectively).

To validate the differences observed in motor coordination and balance, the pole test was employed. Mice in the PD group showed a significant delay in both the time to orient downward on the pole (T-turn time: Control = 13 ± 1; BoNT = 8 ± 1; PD = 21 ± 2; PD + BoNT = 15 ± 1; *** *p* < 0.0001) and the total time to descend compared to the control, BoNT, and PD + BoNT groups (descending time: Control = 25 ± 2; BoNT = 14 ± 1; PD = 35 ± 3; PD + BoNT = 23 ± 1; *** *p* < 0.0001). These findings indicate impairments in motor planning, postural control, and a clear manifestation of bradykinesia in PD. Notably, BoNT-treated mice showed improved performance in the pole test, with significantly shorter T-turn and descending time compared to the control, PD, and PD + BoNT groups. Furthermore, the PD + BoNT group exhibited a marked reduction in both T-turn and descending times compared to the PD group, indicating that BoNT treatment alleviates PD-related motor deficits (Figure 4).

### 3.4. BoNT Diminished Anxiogenic Symptoms in MPTP-Induced Experimental Mice in the Light and Dark Box Test

**Figure 5:**
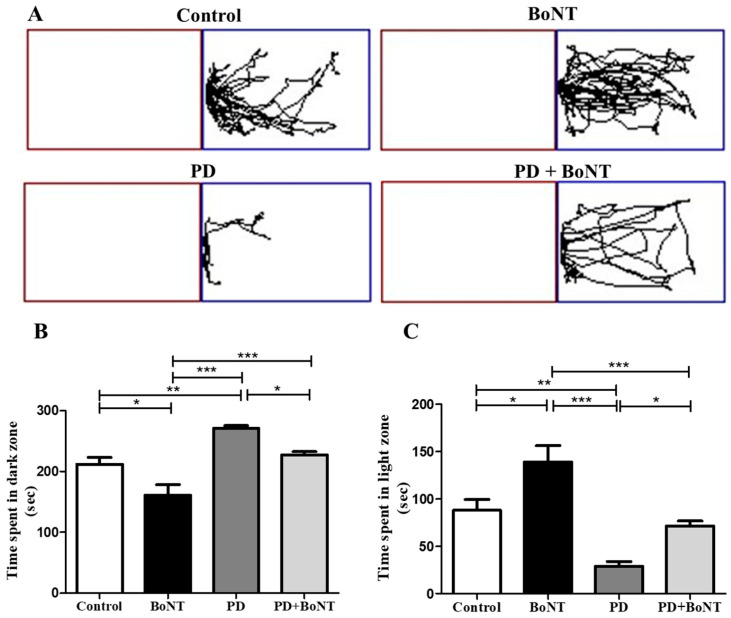
BoNT reduces preference-based anxiety-like behavior in LDBT in MPTP-induced experimental mice. (**A**) Representative tracking image of a mouse during LDBT from Control (*n* = 9), BoNT (*n* = 9), PD (*n* = 9), and PD + BoNT (*n* = 9). The dark zone is marked by red box and the light zone is marked by blue box. The bar graph represents (**B**) time spent in the dark zone, and (**C**) time spent in the light zone by the experimental mice. Statistical significance was determined with one-way ANOVA and further analyzed by Tukey’s post hoc test for multiple comparisons (*, **, *** indicates *p* value ≤ 0.05, ≤0.01, and ≤0.001, respectively).

Further, anxiety-like responses in the experimental mice were assessed using the LDBT. During the test, mice in the PD group exhibited a marked preference for the dark compartment over the light compartment, indicating heightened anxiety behavior (dark compartment: Control = 212 ± 11; BoNT= 161 ± 17; PD = 271 ± 5; PD + BoNT = 228 ± 5, *** *p* < 0.0001). In contrast, BoNT-treated mice spent significantly more time in the light compartment compared to the control, PD, and PD + BoNT (light compartment: Control = 88 ± 11; BoNT = 139 ± 17; PD = 29 ± 5; PD + BoNT = 72 ± 5, *** *p* < 0.0001). Notably, the time spent in the light compartment by mice in the PD + BoNT group was significantly increased than the PD group. The results of LDBT indicate that BoNT treatment reduces anxiogenic symptoms in the MPTP-induced experimental mouse model of PD (Figure 5).

### 3.5. BoNT Decreased Acrophobic-Related Anxiety-like Behavior in MPTP-Induced Experimental Mice in the Elevated Plus Maze Test

During the EPM test, the number of attempts to enter and the time spent in the OAs and CAs by the experimental mice were analyzed. Entries into the CAs by experimental mice are regarded as natural habituation and a reflection of motor behavior, whereas entries into the OAs and the time spent there are indicative of reduced anxiety. Among the experimental groups, BoNT-treated mice showed significantly higher entries into both the CAs and OAs compared to the Control, PD, and PD + BoNT groups. In contrast, PD animals predominantly entered the CAs more frequently than the OAs, reflecting their preference for the CAs. Although the control and PD + BoNT groups showed more entries into both the OAs and CAs compared to the PD group, the frequency of these attempts was nearly equal. However, in terms of duration, mice in the control, BoNT, and PD + BoNT groups displayed a more preference for exploring the OAs over the CAs than that of the PD group (entries in CAs: Control = 9 ± 1; BoNT = 14 ± 1; PD = 5 ± 1; PD + BoNT = 11 ± 1; *** *p* < 0.0001) (entries in OAs: Control = 7 ± 1; BoNT = 12 ± 1; PD = 3 ± 1; PD + BoNT = 8 ± 1, *** *p* < 0.0001).

**Figure 6:**
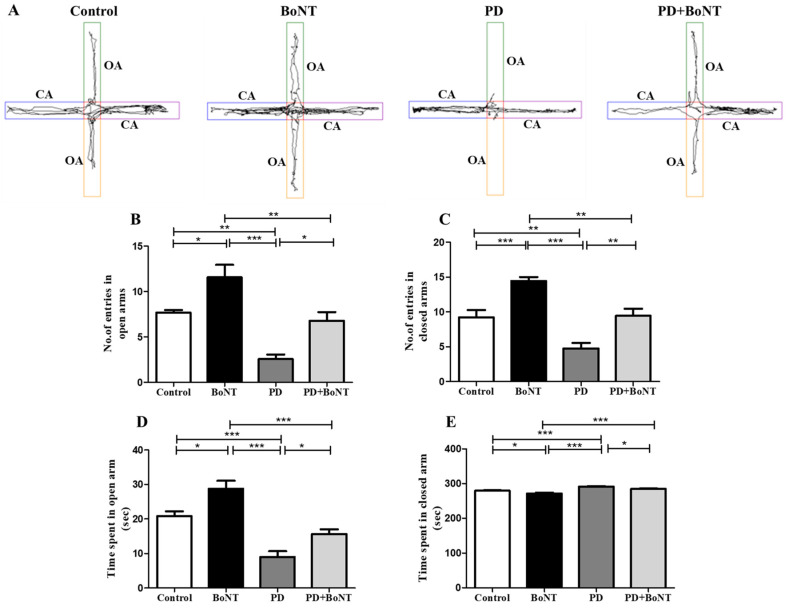
BoNT reduces acrophobia-related anxiety behavior in EPM test in MPTP-induced experimental mice. (**A**) Representative tracking image of a mouse during EPM from Control (*n* = 9), BoNT (*n* = 9), PD (*n* = 9), and PD + BoNT (*n* = 9). The closed arms (CAs) are marked by blue and purple and the open arms (OAs) are marked by green and yellow. The bar graph represents (**B**) number of entries in OAs, (**C**) number of entries in CAs, (**D**) time spent in OAs, (**E**) time spent in the CAs by the experimental mice. Statistical significance was determined with one-way ANOVA and further analyzed by Tukey’s post hoc test for multiple comparisons (*, **, *** indicates *p* value ≤ 0.05, ≤0.01, and ≤0.001, respectively).

As a reflection, mice in PD group spent significantly more time in the CA compared to the control group, BoNT, and PD + BoNT groups. indicating heightened anxiety-like behavior. Whereas BoNT-treated mice spent more time exploring the OAs compared to the control, PD, and PD + BoNT groups. Notably, the PD + BoNT group showed an increased duration in exploring the OAs compared to the PD group (duration in OAs: Control = 21 ± 1; BoNT = 29 ± 2; PD = 9 ± 2; PD + BoNT = 16 ± 1, *** *p* < 0.0001) and (duration in CAs: Control = 279 ± 1; BoNT = 271 ± 2; PD = 291 ± 2; PD + BoNT = 284 ± 1, *** *p* < 0.0001). Thus, the results corroborate with OFT that BoNT improved motor function and alleviates anxiety-like behavior in MPTP-induced experimental mice (Figure 6).

### 3.6. BoNT Protects Dopaminergic Neurons and Reduces the Number of Microglia in the Substantia Nigra in MPTP-Induced Experimental Mice

**Figure 7:**
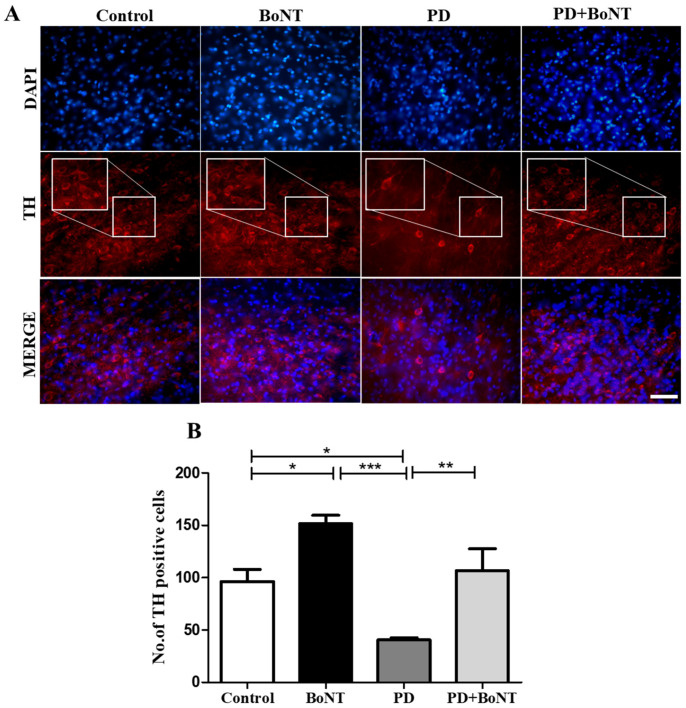
BoNT provides neuroprotection of TH-positive cells in the SN of the experimental mice. The images represent (**A**) fluorescence microscopic images of DAPI, TH staining, and merger of the same in the SN region of Control (*n* = 6), BoNT (*n* = 6), PD (*n* = 6), and PD + BoNT (*n* = 6). (**B**) The bar graph represents the number of TH-positive cells in the SN of experimental mice. Statistical significance was determined with one-way ANOVA and further analyzed by Tukey’s post hoc test for multiple comparisons. (*, **, *** indicates *p* value ≤ 0.05, ≤0.01, and ≤0.001, respectively). Magnification: 400× and scale bar = 50 µm.

To assess the neuroprotective effect of BoNT, the number of TH immunopositive dopaminergic neurons was determined in the SN. The quantitative assessment revealed that the number of TH-positive cells was significantly increased in the BoNT-treated mice when compared to the control, PD, and PD + BoNT groups. PD + BoNT showed an increased number of TH-positive cells in SN when compared to the PD group (Control = 96 ± 12; BoNT = 152 ± 8; PD = 41 ± 2; PD + BoNT = 107 ± 21, *** *p* < 0.0001). Therefore, results show that BoNT restores dopaminergic neurons in SN in the MPTP-induced experimental mice (Figure 7).

**Figure 8:**
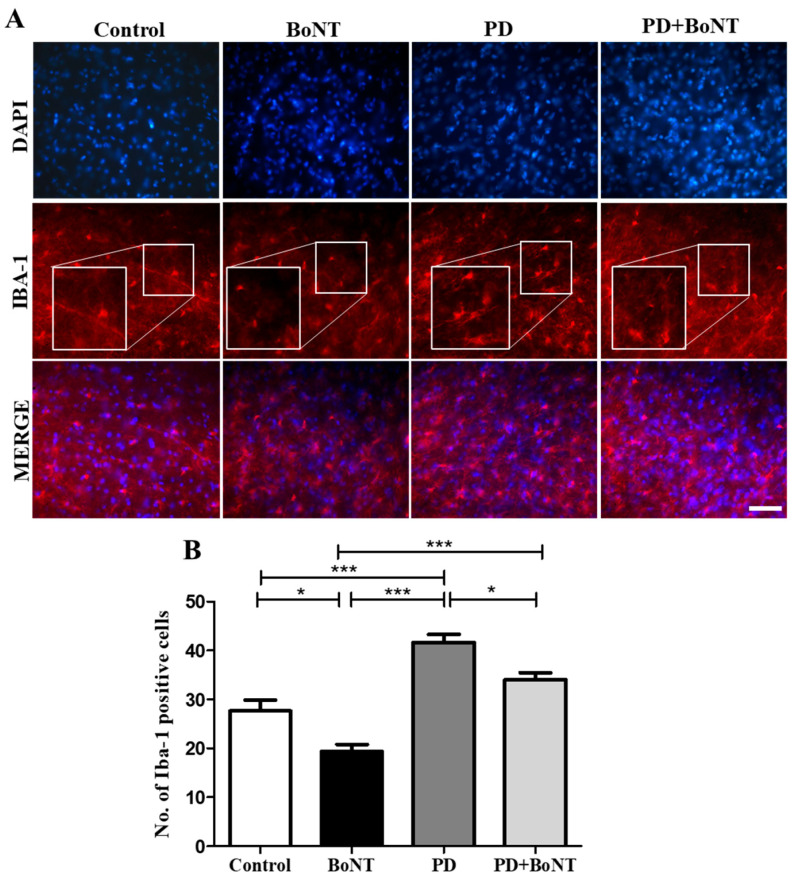
BoNT reduces the number of Iba-1 positive microglial cells in the SN of the experimental mice. The images represent (**A**) fluorescence microscopic images of DAPI, Iba-1 staining, and merge of the same in the SN region of Control (*n* = 6), BoNT (*n* = 6), PD (*n* = 6), and PD+ BoNT (*n* = 6). The bar graph represents (**B**) the number of Iba-1 positive microglial cells in the SN region of experimental mice. Statistical significance was determined with one-way ANOVA and further analyzed by Tukey’s post hoc test for multiple comparisons (*, *** indicates *p* value ≤ 0.05 and ≤0.001, respectively). Magnification: 400× and scale bar = 50 µm.

The immunolabeling analysis of Iba-1 to assess microglia in the SN region revealed that the number of Iba-1-positive microglial cells was significantly reduced in BoNT-treated mice compared to control, PD, and PD + BoNT groups. Notably, the PD + BoNT group also showed a reduction in the number of Iba-1 positive microglial cells compared to the PD group (Control: 28 ± 2; BoNT: 19 ± 1; PD: 42 ± 2; PD + B oNT: 34 ± 1, *** *p* < 0.0001). These results suggest that BoNT treatment reduces microglia-mediated neuroinflammation in the SN of MPTP-induced experimental mice (Figure 8).

## 4. Discussion

Anxiety in PD frequently intersects with motor, cognitive, and emotional symptoms, posing major challenges for accurate diagnosis and effective treatment. Neuroinflammation arising from microglia is widely recognized as a contributing factor to the neuropathogenic progression of PD [32]. Recent studies have highlighted a potential association between neuroinflammation, driven by activated microglia in the brain, and the development of anxiety [33]. Notably, in the PD subjects, the SN exhibits a significantly higher density of microglial cells than other regions of the brain [15,34]. This observation, combined with the finding that neurons in SN are significantly more susceptible to activated microglial-mediated neuroinflammatory changes in PD [35]. Numerous experimental studies have demonstrated microglial activation in PD; however, evidence linking this activation to anxiety remains limited. An increase in the binding of a particular positron emission tomography (PET)-based biomarker for activated microglia, 1-(2-chlorophenyl)-N-methylpropyl)-3 isoquinoline carboxamide ([11C]PK11195) was observed in the striatum and SN along with the retrograde damage to the nigral dopamine neurons in a 6-hydroxydopamine (OHDA) rat model of PD [36]. This indicates that neuronal death can be triggered by inflammatory responses mediated by microglia in PD. Another PET-based finding by Ouchi et al. revealed an increased accumulation of [11C](R)-PK11195 in the midbrain of early PD patients compared to age-matched healthy people [37]. Ample studies have proven that activated microglia are associated with the degeneration of dopaminergic neurons in PD [38,39]. For instance, both in vivo and in vitro studies have demonstrated that lipopolysaccharide (LPS)-induced microglial activation leads to a progressive loss of dopaminergic neurons, thereby contributing to PD-like pathology [40]. Few endogenous peptides and environmental triggers, such as paraquat, substance P, maneb, diesel exhaust particles, and rotenone, cause microglia-mediated neurotoxicity [38,41,42]. Post-mortem investigations of PD brains have confirmed widespread microglial overactivation, particularly in the SN [43]. Elevated levels of neuroinflammatory molecules have been shown to exacerbate oxidative damage, leading to further neuronal loss in PD [44]. Additionally, defects in dopaminergic transmission have been associated with anxiety [45]. Experimental evidence suggests that reduced levels of dopamine and serotonin may increase susceptibility to affective disorders [46]. Accordingly, MPTP-induced anxiety may be associated with dopaminergic neuronal loss in conjunction with disturbances in the serotonergic system in PD [47]. Tadaiesky et al., reported that three weeks following surgery, 6-OHDA-lesioned animals drastically reduced the percentage of entries in the OAs of EPM, exhibiting anxiety-like behavior [48]. Based on a study by J.C.F. Vieira and colleagues, experimental rats with bilateral intranigral injections of 6-OHDA exhibited anxiety-like behaviors in both the EPM and contextual fear conditioning (CFC) tests [49]. Concerning the human situation, N.N.W. Dissanayaka et al., examined anxiety disorders in 79 PD patients using the diagnostic and statistical manual of mental disorders (DSM)-IV criteria and reported that 14% of PD subjects had a comorbid depressive disorder with anxiety [50]. More recently, Negrè-Pagès et al. implemented the hospital anxiety and depression Scale (HADS) to evaluate anxiety and depressive symptoms in 450 ambulatory non-demented PD patients and 98 patients with other disorders [51]. Their findings indicated that patients with possible anxious signs were more common in PD patients (51%) than in the other patients (29%). Therefore, in the present study, the anxiety levels noticed in MPTP-induced experimental animals corroborate the previous finding on anxiety in PD. Thus far, PD research has focused on developing drugs to prevent neurodegeneration, but the existing treatment options have been largely ineffective due to the complex pathomechanisms involved, along with non-motor symptoms, including anxiety. Therefore, drug-based therapies aimed at regulating neuroinflammation could be a potential strategy to alleviate both anxiety and neurodegeneration in PD. This dual approach could provide significant benefits in managing the full spectrum of symptoms associated with PD.

In the aforementioned context, this study focused on investigating the potential of BoNT as a treatment for motor deficits and anxiety symptoms by assessing behavioral outcomes and examining its association with the density of TH-positive dopaminergic neurons and microglia in SN of the MPTP-induced experimental model of PD. The results demonstrated that MPTP-induced motor deficits and anxiety-like behaviors were associated with a reduction in dopamine-positive neurons and an increase in microglial number. BoNT administration mitigated motor and anxiety-like behaviors, protected against dopaminergic neuronal loss, and reduced microglial activation in the SN. These findings validate the therapeutic potential of BoNT in promoting neuroprotection and attenuating microglial burden, thereby contributing to its overall therapeutic benefit in PD. Similarly, Li et al. showed that BoNT reduced depressive-like behavior in a reserpine-induced PD model by suppressing hippocampal microglial activation [52]. These findings align with the present study, suggesting that BoNT may reduce anxiety-like behavior through its anti-inflammatory effects and modulation of central neurotransmission. Eventually, the BoNT-mediated changes observed in the SN can be expected to restore the balance of dopamine levels, thereby alleviating anxiety and complementing improvements in motor behavior. Additionally, the study observed an increased number of TH-expressing cells in the SN region of BoNT-treated animals, which may contribute to reduced anxiety-like behavior. TH is a rate-limiting enzyme responsible for converting L-tyrosine into L-3,4-dihydroxyphenylalanine (DOPA), a key precursor of dopamine in the brain [53]. TH is a bona fide marker for dopaminergic neurons, especially in PD, because loss of TH-positive neurons in SN is a hallmark of PD pathology [54,55]. However, the link between the BoNT-mediated molecular changes and expression of TH in the brain remains unknown. Some animal studies suggest that BoNT undergoes retrograde transport and can alter the neurotransmitter systems such as dopamine, serotonin, and GABA, all of which are critical in anxiety and mood regulation, which could be associated via TH-mediated generation of L-DOPA [56,57,58]. Notably, L-DOPA also serves as a precursor for catecholamine synthesis and norepinephrine production, which are known to play a key role in emotional regulation and stress response also positively contribute to the serotonin system [58,59,60]. While reduced levels of dopamine and serotonin have been linked to anxiety-related symptoms, an increased number of TH-positive cells could contribute to restoring these neurotransmitters and alleviating anxiety as well as other symptoms. Therefore, restoration of neurotransmitter balance may help to promote anxiolytic effects in PD and enhanced TH immunoreactivity may represent a compensatory neurochemical mechanism underlying the behavioral improvements observed following BoNT administration. Moreover, reduced dopamine levels in PD can lead to increased ACh activity, which in turn contributes to motor, cognitive, and anxiety-related symptoms. Considering the direct influence of BoNT on ACh release, the rectification of this neurotransmitter imbalance could be directly linked to its classical mechanism of action.

Moreover, the increased locomotor activity and improved motor coordination observed in the behavior experiments could be expected to contribute to the reduced anxiety levels seen in BoNT-treated animals. A recent report shows that metabolic risk factors such as elevated body mass index, hyperglycemia, and dyslipidemia can reduce the efficacy of deep brain stimulation for non-motor symptoms in PD, while sparing motor benefits [61]. Adding to this, BoNT-mediated improvement in motor function, along with positive metabolic changes, could also be linked to microglial modulation and neuroprotection, thereby mitigating anxiety. However, the link between motor function and anxiety is complex, and there is evidence that anxiety may also be associated with hippocampal plasticity independent of motor areas. In particular, ongoing neurogenesis has been linked not only to learning and memory but also to emotional and anxiety-related behaviors. Recently, we demonstrated that BoNT treatment increases neurogenesis in these experimental animals, thereby improving learning and memory [30]. As reduced hippocampal neurogenesis, reported in many neurodegenerative disorders, including PD, has been associated with anxiety, the increased neurogenesis observed following BoNT treatment might also contribute to the reduced anxiety noted in the behavioral experiments [8,62]. Our previous study demonstrated anxiolytic-like effects of BoNT in aging mice, which were associated with enhanced activity of antioxidant enzymes in the brain [28]. The increased antioxidant levels could contribute to hippocampal plasticity, provide protection against neurodegeneration, which could also represent defensive mechanisms against anxiety.

Moreover, in an independent unpublished study, we demonstrated that BoNT treatment reduced COX-2 expression in aging experimental animals. Increased COX-2 expression in the brain, particularly in regions such as the hippocampus and SN, is a hallmark of neuroinflammatory responses in PD [63]. COX-2 overactivity promotes the production of pro-inflammatory prostaglandins, which can impair synaptic plasticity and neurogenesis but also upregulate inducible nitric oxide synthase (iNOS), leading to excessive NO production [64]. Elevated NO levels, especially when combined with ROS, result in the formation of peroxynitrite, a potent neurotoxin capable of damaging dopaminergic neurons, disrupting neurotransmitter homeostasis, and exacerbating both motor and non-motor symptoms [65]. Excessive NO interacts with superoxide anions, resulting in the formation of reactive nitrogen species (RNS) that cause significant cellular damage and ultimately lead to the death of dopaminergic cells [66]. A study by Kim and colleagues showed that BoNT inhibits the generation of NO and tumor necrosis factor (TNF)-α in RAW264.7 macrophages stimulated by LPS by preventing the activation of c-Jun N-terminal kinase (JNK), Extracellular signal-regulated kinase (ERK), and p38 mitogen-activated protein kinase (MAPK) [67]. It is evident that BoNT inhibits the release of pro-inflammatory cytokines by modulating levels of interleukin (IL)-1β, IL-6, and TNF-α in MPTP and 6-OHDA-induced animal models of PD [68]. These factors appear to predominantly originate from microglial activation or other cellular sources in the brain, both of which can contribute to neurodegeneration and suppression of neurogenesis, potentially serving as underlying mechanisms of motor disorders and anxiety. Therefore, future studies need to incorporate detailed neuroinflammatory profiling to evaluate BoNT-mediated effects, including changes in TNF-α, IL-1β, and IL-6 expression, either in the brain or under in vivo and in vitro conditions using different PD models and cell lines of neuronal and non-neuronal origins. In a pilot experiment using brain extracts of BoNT-treated PD animals, we observed a trend toward reduced nitrite levels with the Griess assay (data not shown), indicative of decreased NO and nitrosative stress. However, the effect of BoNT on NO levels remains insufficiently understood, highlighting the need for further comprehensive studies to elucidate its mechanisms and therapeutic potential. Taken together, the present study not only validates previous findings but also extends them by providing valid clues that BoNT exhibits anti-microglial activity, which may partly contribute to neuroprotection in the SN of MPTP-induced experimental models of PD. Apart from these factors, PD appears to differentially affect males and females. While estrogen may provide partial neuroprotection against neurodegeneration, males are generally more vulnerable to PD. However, the prevalence of PD among females remains substantial. Since this study included only male animals and the observed effects may not fully translate to females, future research should incorporate female subjects to better address potential sex-specific differences. Moreover, it is important to note that the observed associations between BoNT treatment and reduced anxiety are based on behavioral outcomes. Therefore, establishing a direct causal relationship between PD and anxiety requires further investigation. Despite its relatively short half-life, BoNT can produce effects that extend well beyond its initial presence in the system [69]. While the endurance of BoNT-mediated clinical effects can last from 6 months to 1 year, further experimental validation is needed to confirm its long-lasting efficacy over extended periods, and the possibility of dose-related adverse effects cannot be completely excluded [17,18].

## 5. Conclusions

This study demonstrates that BoNT treatment alleviates motor impairments and reduces anxiety-like behaviors in a PD mouse model. The outcome of the result highlights novel and comprehensive insights into the mechanisms underlying its anxiolytic effects of BoNT in a widely used animal model of PD, which appear to involve motor function improvement, modulation of microglial activity, and the pro-neurogenic properties of BoNT. While further studies are required to validate and interpret these findings, the present results qualitatively lay down a strong foundation for understanding the multifaceted therapeutic potential of BoNT in PD. Moreover, the anxiolytic effects observed in this study may be translatable to other disorders in which anxiety-related symptoms are prominent. With careful consideration of optimal dosing and treatment duration, BoNT could be regarded as a promising therapeutic agent for many neurodegenerative disorders.

## Data Availability

All data are provided within the manuscript.
